# Detection of a *TRAF1*-*ALK* fusion in an anaplastic large cell lymphoma patient with chemotherapy and ALK inhibitor-resistant disease

**DOI:** 10.1186/s13104-015-1277-7

**Published:** 2015-07-18

**Authors:** Kasey Lawrence, Brian Berry, John Handshoe, David Hout, Rosetta Mazzola, Stephan W Morris, David L Saltman

**Affiliations:** Insight Genetics, Suite 510, 2 International Plaza, Nashville, TN 37217 USA; Department of Pathology, Royal Jubilee Hospital, 1952 Bay Street, Victoria, BC V8R 1J8 Canada; British Columbia Cancer Agency, 2410 Lee Avenue, Victoria, BC V8R 6V5 Canada

**Keywords:** TNF receptor-associated factor 1 (*TRAF1*), Anaplastic lymphoma kinase (*ALK*), Lymphoma, ALK inhibitor

## Abstract

**Background:**

The anaplastic lymphoma kinase (*ALK*) gene encodes a receptor tyrosine kinase, which was first identified as the fusion partner of the nucleophosmin (*NPM1*) gene in the recurrent t(2;5)(p23;q35) found in a subset of anaplastic large cell lymphoma (ALCL). Several distinct, non-*NPM1*, *ALK* fusions have subsequently been described in lymphomas and other tumor types. All of these fusions result in the constitutive expression and activation of ALK and ALK signaling pathways, ultimately leading to the malignant phenotype.

**Case report:**

A non-*NPM1* fusion partner of *ALK* was identified in a 32-year-old Caucasian male ALCL patient whose disease was refractory to standard chemotherapy and autologous stem cell transplantation, and exhibited a poor response to a first-generation ALK inhibitor. Non-allele-specific *ALK* RT-qPCR revealed ALK overexpression and 5′ RACE PCR revealed that the patient’s lymphoma expressed a *TRAF1*-*ALK* fusion.

**Conclusions:**

We report the case of an ALCL patient whose tumor harbored the newly recognized *TRAF1*-*ALK* fusion and describe the clinical outcome after treatment with an ALK inhibitor. The short survival of our patient may reflect a propensity toward aggressive behavior in lymphomas that express this *ALK* fusion.

## Background

The anaplastic lymphoma kinase (*ALK*) gene was discovered in 1994 as the fusion partner of the nucleophosmin (*NPM1*) gene in the recurrent t(2;5) chromosomal translocation in anaplastic large-cell lymphoma (ALCL) [1]. The NPM1-ALK fusion protein produced by this rearrangement results in constitutive activation of the ALK kinase and is associated with the deregulation of multiple signaling pathways downstream of ALK, culminating in increased cellular proliferation, survival and migration [2]. Since the original description of the *NPM1*-*ALK* fusion gene in ALCL, a large number of distinct *ALK* fusion partners have been identified in ALCL as well as a variety of other malignancies including diffuse large B cell lymphoma, inflammatory myofibroblastic tumor, non-small cell lung cancer, and renal cell carcinoma [2, 3]. Patients with ALK-positive ALCL treated with ALK inhibitors typically have high response rates and durable progression-free survivals, even if they have been heavily pretreated [4, 5].

Recently, a novel fusion was described between the TNF receptor-associated factor 1 (*TRAF1*) gene and *ALK* in an ALCL patient who experienced an almost 3-decade history characterized by several disease relapses followed ultimately by apparently successful treatment with high-dose chemotherapy and autologous stem cell transplantation [6]. In this report we describe an additional ALCL patient whose tumor contained a *TRAF1*-*ALK* fusion. Unlike the case described by Feldman et al. [6], our patient had aggressive disease characterized by poor responses to chemotherapy, autologous transplantation, a CD30 antibody–drug conjugate, and a first-generation ALK tyrosine kinase inhibitor.

## Case report

**Figure 1:**
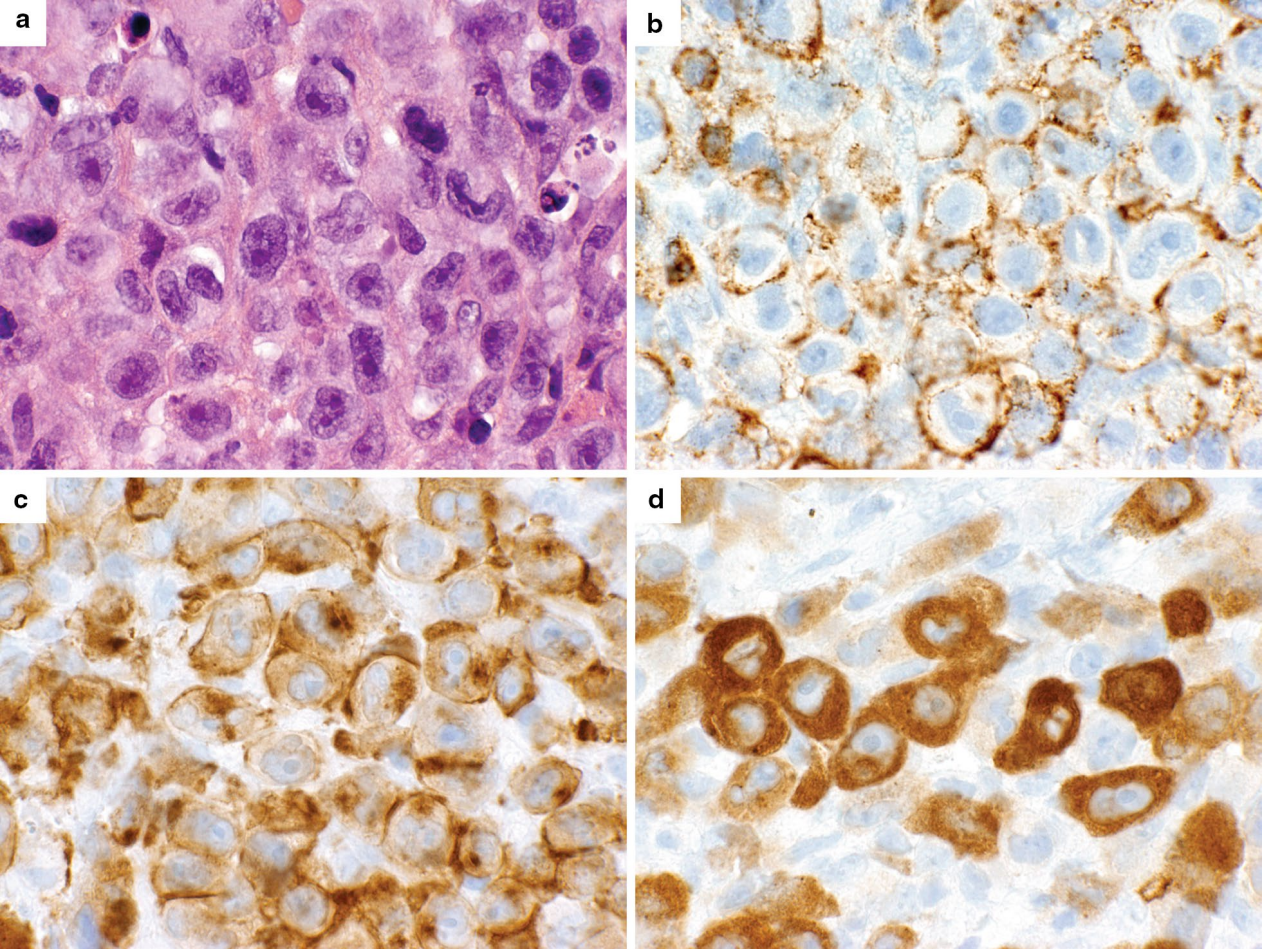
Pathologic findings in the lymph node biopsy. **a** Diffuse infiltration of large and pleomorphic lymphocytes (H&E, ×600). **b** Membranous expression of CD43 (×600). **c** Membranous and golgi expression of CD30 (×600). **d** Cytoplasmic expression of ALK (×600).

The patient was a 32-year-old Caucasian male who was diagnosed in 2004 with mycosis fungoides after developing a pruritic rash. There was no family history of cancer. He was initially treated with topical nitrogen mustard and later received UVB narrow band therapy. In October 2012, the patient presented with a cough, dyspnea, fever, chills, left pleuritic chest pain and significant weight loss. These symptoms were associated with a flare of his skin lesions. A CT scan of the chest demonstrated extensive left cervical, supraclavicular and mediastinal adenopathy, as well as left upper lobe airspace disease. Review of pathology from a left cervical lymph node biopsy from December 2012 revealed complete effacement of the normal architecture by a diffuse infiltration of large and giant pleomorphic lymphocytes characterized by conspicuous nucleoli and large amounts of cytoplasm (Figure 1a). Immunohistochemical stains showed weak CD45 and strong CD43 expression (Figure 1b), with strong CD30 expression in the membrane and Golgi patterns (Figure 1c). The malignant cells expressed cytoplasmic ALK (Figure 1d) but lacked expression of CD 3, 4, 5 and 8. A T cell lineage was confirmed in the tumor cells of the initial biopsy sample by clonal T-cell receptor gamma gene rearrangement. Identical infiltrates were identified in material from recurrent biopsies of skin and subcutaneous tissue, and from lung and endobronchial biopsies at the time of progression.

**Figure 2:**
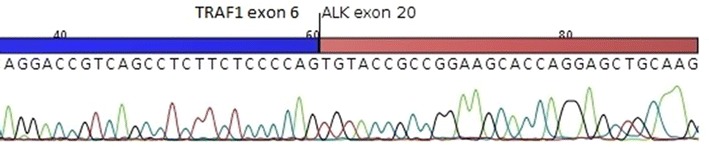
Sanger sequencing electropherogram of the *TRAF1* exon 6-*ALK* exon 20 fusion junction. The sequences in *blue* are from exon 6 of the *TRAF1* gene. The sequences in *red* are from exon 20 of *ALK*. The *TRAF1*-*ALK* fusion junction in this case is identical to that previously reported in the literature by Feldman and colleagues [6] and Abate et al. [7].

After an initial response to four cycles of cyclophosphamide, doxorubicin, vincristine and prednisone (CHOP) chemotherapy, the disease progressed rapidly while the patient was on therapy. He was started on gemcitabine, dexamethasone and cisplatin (GDP) chemotherapy in preparation for an autologous stem cell transplant but progressed after 3 cycles. The patient then received one cycle of the CD30 antibody–drug conjugate brentuximab, with a response but tolerated the treatment poorly. He was subsequently started on the ALK small-molecule kinase inhibitor crizotinib and tolerated the therapy well with an improved performance status. A fluoro-deoxyglucose positron emission tomography (FDG-PET) scan performed after 3 months of crizotinib showed an improvement in the previously noted airspace disease in the left lung but an enlarging FDG-avid 5.2 cm mass in the apex of the left lung. There was no evidence of disease outside the chest. A CT scan-guided biopsy of this mass was positive for ALK by immunohistochemistry and RT-qPCR using an *ALK* RGQ RT-PCR Kit (Qiagen, Hilden, Germany). The remaining RNA from the lymph node biopsy was reverse transcribed to cDNA and used in a 5′ RACE PCR followed by Sanger sequencing to identify the *ALK* fusion partner. Analysis of sequence data revealed a *TRAF1*-*ALK* fusion in which 3′ end of exon 6 of *TRAF1* was fused to the 5′ end of exon 20 of *ALK* (Figure 2). The mRNA fusion junction in *TRAF1* and *ALK* in the case was identical to that present in the *TRAF1*-*ALK*-positive ALCL case reported by Feldman et al. [6].

The patient underwent an unrelated-donor stem cell transplant in December 2013 but disease recurred in the left lung within 3 months post-transplant, and failed to respond to the reintroduction of crizotinib and brentuximab. Endobronchial biopsies from lesions in the left and right lungs revealed ALK-positive ALCL. The patient died of progressive lymphoma 6 months post-transplant.

Molecular testing of the patient’s biopsy samples was performed with the consent of the patient using a study assay approved by the BC Cancer Agency Research Ethics Board (H13-01763) and conducted in accordance with the ethical principles of the Declaration of Helsinki.

## Conclusions

We describe an additional case of *TRAF1*-*ALK* fusion in ALCL. Unlike the initial case, which was characterized by a nearly 3-decade course punctuated by multiple therapy-responsive relapses [6], our patient had a short survival after responding poorly to chemotherapy, autologous stem cell transplantation, CD30 antibody–drug conjugate therapy, and the first-generation ALK inhibitor crizotinib.

Of note, while this case report was undergoing peer review an independent study of two additional cases of *TRAF1*-*ALK*-expressing ALCL was published [7]. Interestingly, these two cases had very different clinical courses: one was a chemotherapy refractory patient whose tumor rapidly progressed to a leukemic phase and contained additional genetic abnormalities (*TP53* loss, *cMYC* amplification, *PRDM1/Blimp1* loss) that may have contributed to the aggressiveness of the malignancy while the second was an 11-year-old whose ALCL lacked these genetic abnormalities and who had a remarkable anti-tumor response to conventional chemotherapy and remains in clinical remission. This study also presented experimental data suggesting that the activation of NFκB signaling—mediated by the interaction of TRAF1-ALK with TRAF2, which in turn modulates CD30-mediated activation of NFκB—contributes to oncogenesis associated with TRAF1-ALK [7].

Whether the natural histories observed for these cases are related at least in part to the presence of *TRAF1*-*ALK* rather than the more common *NPM1*-*ALK* fusion is unclear given the limited clinical experience with *TRAF1*-*ALK*-positive ALCL. Preclinical analysis has suggested that various *ALK* fusions that differ only in the identity of their partner genes respond with differential sensitivity to structurally diverse ALK kinase inhibitors [8]. However, it is not yet clear if these preclinical differences are of clinical relevance.

Nevertheless, some *NPM1*-*ALK*-positive ALCL patients who have progressed after multiple prior therapies have been shown to respond favorably to crizotinib [4, 5]. ALK-inhibitor resistance-causing mutations have been detected in *NPM1*-*ALK*-positive ALCL patients who have not responded to ALK inhibitors [4]. Interestingly, some of the *NPM1*-*ALK*-positive ALCL patients who failed to respond to crizotinib have been shown to harbor inhibitor resistance mutations after only brief (1–2 month) treatment with the kinase inhibitor, suggesting the presence of the mutations de novo (i.e., prior to inhibitor therapy) [4]. Our patient may have had one or more primary resistance-causing mutations at diagnosis or developed ALK inhibitor resistance after multiple lines of cytotoxic chemotherapy. In addition, however, the patient’s failure to achieve a sustained response to cytotoxic therapy, a CD30 antibody–drug conjugate or an ALK inhibitor suggests the presence of one or more alternative drug resistance mechanisms in addition to the possible presence of ALK inhibitor resistance mutations.

## Consent

Written informed consent was obtained from the patient’s next-of-kin (mother) for publication of this Case report and any accompanying images. A copy of the written consent is available for review from the Editor-in-Chief of this journal.

## Abbreviations

TRAF1: tumor necrosis factor receptor associated factor 1
ALK: anaplastic lymphoma kinase
ALCL: anaplastic large cell lymphoma
FDG-PET: fluoro-deoxyglucose positron emission tomography
RACE: rapid amplification of cDNA ends
